# Supplementary material for: Patterns of Intron Gain and Loss in Fungi
Source: PLoS Biol. 2004 Nov 30;2(12):e422. doi: 10.1371/journal.pbio.0020422 (PMC532390; doi:10.1371/journal.pbio.0020422)
Supplement: Table S1 — Also available at http://genes.mit.edu/NielsenEtAl/. (4.3 MB ZIP). [file pbio.0020422.st001.zip › NielsenEtAl/html/1223.html]

AN6844.1.NCU03777.1.MG04012.1.FG07019.1


```
 CLUSTAL W (1.82) Multiple Sequence Alignments - Introns Inserted


Sequence 1: NCU03777.1	508 aa
Sequence 2: FG07019.1	480 aa
Sequence 3: MG04012.1	475 aa
Sequence 4: AN6844.1	481 aa
Alignment Length: 519 aa
Number Identitical Residues: 174 aa
Alignment Score (without introns) 9258


MG04012.1 	-----MPLRAKILSAKPEYR--~-------------------~--~--AQ0MSTVKAQYA
NCU03777.1	MLVSKSIASRAGLASAVASRSC~RPSFTAMPLRAKVLGQQAP0IS~TQAA~AELFKPVEG
FG07019.1 	-----MVQQGKVLSSSLGAR--~-------------------~-Q0VSDE~ATTIKPLPE
AN6844.1  	-----MPLRAKVTNPGFAATSN0-------------------~MS~TASN~PDIPKAQHG
          	              .     :                       .          *.   

MG04012.1 	EDEQDVLFSSLYGLRTIELNRPKKLNSLNLSMIKKITPRLL~EWEKSDMANVIVMKGAGD
NCU03777.1	DEPEDVLFNSLYNLRSVELNRPAKYNALNGSMIRKIAPRLL~EWERSDMANVIVIKGSGE
FG07019.1 	DEPNDVVFESKYGLRTMMLNRPQKLNSLNSSMIRKMVPRLV~EWEKSDLANVVVLKGAGH
AN6844.1  	DEPDDVLFNSLFGVRLVELNRPKKLNSLNGSMVRKILPRLK0EWEKSQLANIIMVAGAGT
          	:: :**:*.* :.:* : **** * *:** **::*: ***  ***:*::**:::: *:* 

MG04012.1 	KAFCAGGDVAALAEANVKGKQGVQQSAAYFAEEYALDHLIATYQTPYIAFMDGITMGGGV
NCU03777.1	KAFCAGGDVAALAKQNAEGPEGVKKSVDYFGLEYKLNHLISTYTRPYVAFLDGITMGGGV
FG07019.1 	KALCAGGDVAALAEMNQQNETGWMRSSAYFALEYKLDHYIATYNKPYIAFMDGITMGGGV
AN6844.1  	KALCAGGDVAALALQNEKGPEGQQASTDFFGLEYKLDHVIATYSKPFISVMDGITMGGGV
          	**:**********  * :.  *   *  :*. ** *:* *:**  *:::.:*********

MG04012.1 	GLSAHAPMRIATERTVFAMPETNIGFFPDVGASFFLPRLDGAIGTYLGLTGETLSGANVF
NCU03777.1	GLSIHAPFRIATERTVFAMPETKIGFFPDVGASFFLPRMPGQVGPYLGLTSALLKGVQVY
FG07019.1 	GLSAHAPFRIATENTVFAMPETGIGFFPDVGASFFLPRMNGSIGTYLALTSAQLRGPNVF
AN6844.1  	GLSVHAPFRIATERTVFAMPETTIGFFPDVGGSFFLPRLDGEIGTYLALTSARLTGVQAL
          	*** ***:*****.******** ********.******: * :*.**.**.  * * :. 

MG04012.1 	YTGIATHYMHSTTLPLLESRLAELRFNDYDSMQERLAHITTAIEEYTTGLPHDQPILLAG
NCU03777.1	YAGIATHYLHSSSLPALESRLAELTPRDYWTIEQRLSVINDTIEEFSTGVPYDENIEIGG
FG07019.1 	YAGIATHYLHSTSLPDLEARLAELRFRDSDTLPERLALINQTLEEFCTGLPHDQPIELTG
AN6844.1  	YAGIATHYFDSSVLGNLTQRLSELVFRDSATLQERLDLINRTMAEFATGLPEEP--QLAG
          	*:******:.*: *  *  **:**  .*  :: :**  *. :: *: **:* :    : *

MG04012.1 	ELRQAIDRCFSHDSVAKIVAALQNEAES---GPVREWAQKTLDTLHSRSPISLHVSLRQL
NCU03777.1	KIRLAIDRCFKYDKIDEIIAALKEEAAEGAKGGVQSWAKNTLEELTQRSPTSLHVTLRQM
FG07019.1 	AVRQAIDRCFNRHTITEIIAALKAEKGN---SSTRGWARQQLETLHKRSPTALHVALRQM
AN6844.1  	QLRSAIDRCFRHDTVEQIMKALEREKKC------KKWAQETLETMSQRSPTSLKVALRQM
          	 :* ******  ..: :*: **: *         : **:: *: : .*** :*:*:***:

MG04012.1 	RIAQKWGIRETFEKEHQLAAKFMASPDFNEGVTARLISKPKRNP-NYNPAKIGDVDTESE
NCU03777.1	RLGKSWGIAHTFKREHQMAAKFMKSHDFNEGVTALLIDKGANGPAKWKPASLDEIPPGAN
FG07019.1 	RIGGEWDIAETFKREHQIATKFMQHHDFTEGVSALLIRKNAP---VWEPESLAAIG-GTN
AN6844.1  	RVGQAWGIRETFQREYEIAARFMQHPDFVEGVKARLMSKPPRQA-SWQPATLAEVS--EK
          	*:.  *.* .**::*:::*::**   ** ***.* *: *    .  ::* .:  :    :

MG04012.1 	TFKNFFRPAKDLQPIKFLNKRDFNEYPFTDFGLPRERQVRELVQTED----LSQREVISK
NCU03777.1	ISEDYFRNDPEVPVLELLNDRSYMQYPYNKFGLPNDYDVKEAIEKGN----FTREKLIDH
FG07019.1 	VAKPFFEYDDDHP-LHLFTDRTYKEYPHQELGVPTEKEVEKVLSQGT----YTREELANK
AN6844.1  	DVDEFFKIPQGKERIQLLSQENWRSYPHS-YGLPSEKAIEKFIREADPKSRASKGEVIEH
          	  . :*.       :.::... : .**.   *:* :  :.: :    ..:  :: :: .:

MG04012.1 	FVALSKARLGVKEVVEEIVYRKTVAGAHGRATW0LPNIA------
NCU03777.1	FVETRRGKQGVREAVSDVLDRMAVRSKGTEHVQ~WKKE-------
FG07019.1 	IIASRNGRQGISEVVADIIDRKTVVNDKRKAVW~MKDEVAPGSRL
AN6844.1  	FVKEFEHKEGVKEKVAEVLARKTTKSAEG-LIW~QGEGAETDGQ-
          	::   . : *: * * ::: * :. .          .   ...
```
